# Developing a digital archive system for imperial Chinese robe in the Qing Dynasty

**DOI:** 10.3389/fnins.2022.971169

**Published:** 2022-07-28

**Authors:** Miao Su, Saiquan Li, Yan Lu, Limei Yang, Yiting Duan, Kaida Xiao, Michael Pointer, Ming Ronnier Luo, Xiaoxuan Liu

**Affiliations:** ^1^College of Textile Science and Engineering, International Silk Institute, Zhejiang Sci-Tech University, Hangzhou, China; ^2^International Centre for Silk and Silk Road Studies, Hangzhou, China; ^3^School of Design, University of Leeds, Leeds, United Kingdom; ^4^College of Optical Science and Engineering, Zhejiang University, Hangzhou, China

**Keywords:** digital archive system, color characterization model, imperial silk robe, “Qianlong Palette” color chart, color image reproduction

## Abstract

The digital archive of cultural heritage provides new opportunities for the protection of the cultural heritage and the development of online museums. One of the essential requirements for the digitization is to achieve accurate color reproduction. Taking the Imperial Chinese robes in the Qing Dynasty as an example, this study aims to develop a digital achieve system to digitize the robes using a high-end imaging system and accurately reproduce their color properties on a display. Currently, there has been very limited study focused on the color reproduction of silk fabrics or other textile materials. The conventional color management process using a traditional color chart, however, may not be suitable for the reproduction of silk fabrics because they have very high gloss. To address this difficulty, a unique “Qianlong Palette” color chart, consisting of 210 silk fabric samples, has been specifically produced for optimizing the color reproduction of silk fabrics and a color image reproduction system has been developed for the digitization and archiving of the clothing fabric for the royal court. Color characterization models using both the “Qianlong Palette” color chart and the traditional color chart, and different mapping methods, are compared and the model with highest accuracy used in a self-programmed interface for automatically processing textile images in the future. Finally, the digital archive system has been validated using six garments of silk fabric relics. The color differences after the color image reproduction are all less than 3.00ΔE*_ab_, indicating acceptable color reproduction of the system. The images after color reproduction have also been evaluated subjectively by experts from the museum and the results are considered satisfactory. Our results show that the newly designed “Qianlong Palette” color chart exhibits superior performance over the conventional color chart in effectively predicting the color of the silk fabrics. The self-programmed graphical user interface for image color management can serve as a powerful tool to truly reproduce the color of various silk fabric relics in museums in the future and digitally archive those valuable cultural relics for different uses.

## Introduction

With the rapid development of modern science and technology, contemporary museums make use of various digital multimedia technology to realize the presentation and protection of the historical materials and cultural heritage, which enriches the content and form of an exhibition and also improves the interactive experience of the audience (Shi et al., 2008; Hampson et al., 2012). The digital archive of cultural heritage is of great importance not only for aesthetic purposes for online museums and culture communication but also because it provides information about the objects’ provenance, manufacturing technique and protections (Navarrete, 2013). Faithfully reproduction of their visual appearance is one of essential requirements of digitization. Especially for those colorful cultural relics such as the dyed silk fabric, the digital color acquisition and management is of great importance such that an accurate color match between the real silk fabrics and the image is highly desirable (Sarti and Plutino, 2021).

The last feudal dynasty in China, the Qing Dynasty (1644 - 1911) witnessed the great success of natural dyeing techniques. During Qianlong’s reign (1711 - 1799, the sixth emperor in the Qing Dynasty), various natural dyestuffs were used for clothes of silk fabrics and different colors classified into several main dye categories have been strictly linked with the social status (China Silk Museum., 2014). The “Qianlong Palette” refers to the restored colors of the dyeing archive of the mid-Qing Dynasty (Liu, 2020). The “Qianlong Palette” is not only a representation of the typical textile colors of the Qianlong period but also a color restoration of the entire silk dyeing process during the heyday of the Qing Dynasty. Silk was the main material for royal costumes at that time, and silk is one of the fabric materials which possesses the best affinity to most of the natural dyes. The dying techniques using natural plants are still of great significance to the contemporary application of environmental-friendly and sustainable textile dying (Yang et al., 2021). Taking the Imperial Chinese robes in the Qianlong reign, the garments containing the most important color sets from the “Qianlong Palette,” as an example, this study aims to develop a digital archiving system to digitalize the robes using a high-end camera system under controlled lighting conditions and truly reproduce their color properties on a display.

Because the color control of both the digital camera and the display is device dependent, it is difficult to reproduce accurate colors between different media and it is common to see color distortion. The color management process developed to precisely control the color across different media has been successfully applied in the graphic arts industry (Hunt, 2005; Sharma and Bala, 2017). A device color characterization model is frequently used to connect device color space to human visual perception in order to achieve the same appearance on different devices *via* a transformation between two color spaces, e.g., for a digital camera the image RGB raw data from the sensor is transformed into CIE color coordinates, for example, XYZ or CIELAB (Green and MacDonald, 2011).

The performance of a color characterization model is affected by both the training dataset and mathematical model (Chou et al., 2013; Liang et al., 2022). For the camera color characterization model, a standard color chart, such as X-rite SG chart, is widely used as the training dataset, while a polynomial regression model is used to predict relationships between camera RGB and CIE XYZ tristimulus values. Although it has been very successful in the graphic arts industry, there has been very limited study focused on the color prediction of textile materials or silk fabrics. Considering that the material properties of the silk fabrics have a very high gloss, and they are obviously different from the uniform color patches on the classic color charts, the conventional color management process may not be suitable for the color reproduction of silk fabrics. Hence, in the present study, effort has been made to restore the colors in the “Qianlong Palette” on silk fabrics using the natural plant dying techniques and to use these samples as the targets for silk fabric color characterization. Moreover, the mathematical mapping methods, such as the polynomial regression with different order, used for transformation also influence the predictive accuracy of the digital imaging system (He et al., 2021). However, it is not known whether conventional aping methods are appropriate for the color reproduction of silk fabrics. This will, which is also aimed to be tested and discussed.

In this study, a color image reproduction system has been developed for the digitization and archiving of the royal silks in the Qing Dynasty. It includes the steps of image acquisition, device color characterization, image processing, and system validation. Both the widely used classic color chart and the self-developed “Qianlong Palette” color chart were used as the training datasets to develop the camera color characterization model with attempt of different mathematical mapping methods. Their performance was compared in terms of the color prediction accuracy of the silk fabrics. In addition, a self-programmed graphical user interface has been developed based on the results for the image processing of any new silk fabrics taken with the digital imaging system to achieve the highest color reproduction accuracy.

## Materials and methods

### Development of the “Qianlong Palette” color chart

**FIGURE 1:**
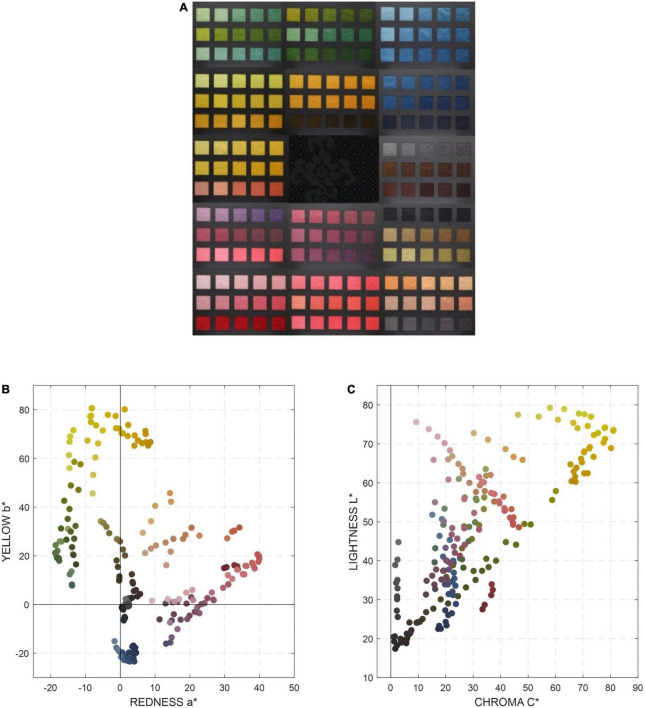
The “Qianlong Palette” color chart **(A)** and the Color distributions of the “Qianlong Palette” color chart in CIELAB color space **(B)** a* b* plane and **(C)** L*C* plane.

Based on archeological documents, forty-two recorded colors were selected in this study, representing the colors of court costumes and accessories in the Qianlong period of the Qing Dynasty (Han et al., 2018; Liu, 2020). A special silk material, plain crepe satin, was selected for dyeing in order to reproduce these royal silks. Nine different natural dyestuffs, including safflower, indigo, sappanwood, phellodendron amurense, sophora flower bud, acorn cup, nutgall, cotinus coggygria, and gardenia, with different dyeing auxiliaries including alum, black alum, alkali, citric acid, and fructose were used according to documented recipes. With each typical color dyed at five levels of dosage (color shade, 40, 70, 100, 130, 160%), altogether 210 silk fabrics samples (4.5 cm × 4.5 cm) were prepared to assemble the new “Qianlong Palette” color chart (see Figure 1A).

For different samples, the dyeing methods (direct dyeing, mordant dyeing, or reduction dyeing), the dyestuffs, the concentration, and the dyeing conditions varied depending on the specific color of the sample. Taking the bright yellow (100% concentration) in the yellow series as an example, the dying process included: prepare 5 g plain crepe satin (made of 100% mulberry silk with the gram weight of 68.59 g/m^2^); heat 7.5 g sophora flower bud and boil in 75 g water; simmer for 30 mins and repeat three times; filter the dye solution; dip the silk fabric sample into the alum mordant solution for 30 mins; dip the sample into the sophora flower bud dye solution and soak at 60°C for 60 min; repeat the dying process three times. After the above preparation, the samples were stored in a dry and dark environment to maintain a good storage condition and prevent yellowing or staining throughout use (Park, 2007). In order to facilitate the subsequent experimental operation, a 4.5 cm × 4.5 cm patch was cut out from each of the 210 dyed plain crepe satin samples and then pasted on to black cardboard to produce the color chart. With the five shades of each color arranged in one row and 3 rows in each card, a total of 14 cards were created as the new “Qianlong Palette” color chart.

The color of each patch in the “Qianlong Palette” color chart was measured by the JETI spectroradiometer. Figures 1B,C show the color distributions of all 210 colors in the “Qianlong Palette” color chart in CIELAB color space. The L* values of all 210 colors ranges from 17.4 to 79.3; a* ranges from −18.7 to 39.9; b* ranges from −23.4 to 80.7. The “Qianlong Palette” covers different color series. Among all the colors, red and yellow are the main color series, which are the most important color representations of the Imperial Chinese robes.

### Framework for the digital archive system

**FIGURE 2:**
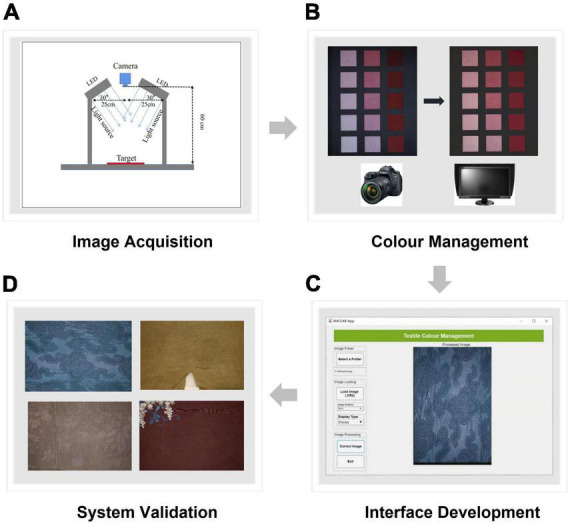
The framework for the digital archive system.

The digital archive system was established in four steps, as shown in Figure 2. The first step was the development of the digital imaging system for image acquisition. The second step was the color management of both the camera and the display. The best color characterization model in this step was chosen for the next step of image processing. In the third step, a self-programmed graphic user interface was developed based on the models for image processing. Finally, the digital system was validated using six representative garments in the Ming and Qing Dynasties and the system accuracy was evaluated both objectively and subjectively. The specific process of each step is explained in the sections below.

#### Digital imaging system for image acquisition

**FIGURE 3:**
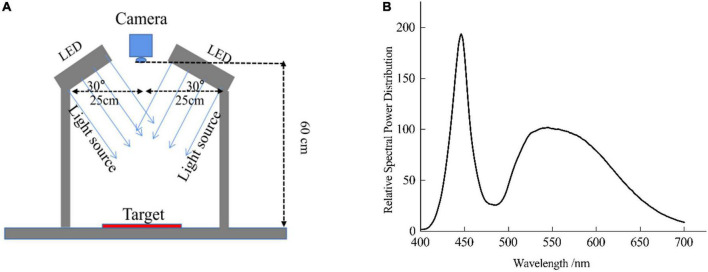
**(A)** The digital imaging system for silk fabric image capture; **(B)** The relative spectral power distribution of the lighting units.

A digital imaging system was developed to capture the images of the silk fabrics, as shown in Figure 3A. The system used a professional copy stand with two LED lighting units (6500K white light, Figure 3B shows the relative spectral power distribution) to provide a well-lit, stable and consistent condition for silk image capturing. Every time before image capture, the lights were turned on for more than 25 min to stabilize. The system was placed in a dark room and there was no other light source in the room while capturing images. A high-resolution digital SLR camera (Canon EOS 5D Mark IV) controlled by specialized software was fixed at the top center of the copy stand and the vertical distance between the camera lens and the target on the stand was 60 cm. Various capture settings were optimized and fixed before the formal capture of the chart and silk fabrics. After testing, the camera parameters were set at: manual mode, ISO200, aperture size F/5.6, shutter speed 1/80s, customized white balance for 6500K, and an image resolution of 6720 (width) * 4480 (height) pixels (3:2 aspect ratio). Camera RAW image file (.CR2 file) without any color correction was used. Comparing with a color processed image by the camera, camera RAW RGB has direct connection of lighting intensity of object (He et al., 2021). The open-source converter *dcraw.exe* was used to decode the RAW image file into a TIFF image and the MATLAB was used to extract the image RGB values.

#### Camera color characterization

**FIGURE 4:**
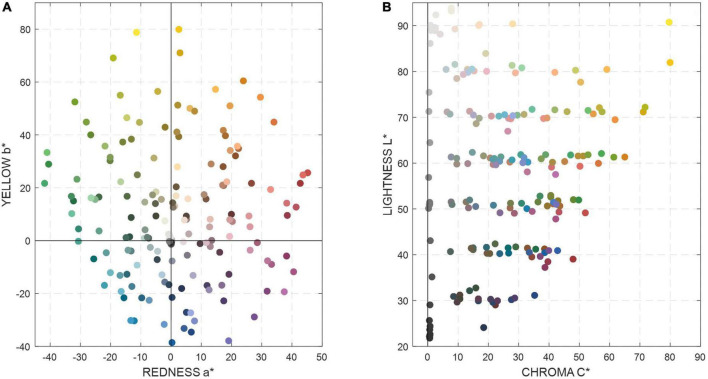
The color distributions of the DC chart (232 matte patches) in CIELAB color space **(A)** a* b* plane and **(B)** L*C* plane.

A camera color characterization model is normally determined by the characterization target (training dataset) and the mapping method used to transform the camera signals, image RGB raw data, into CIE color coordinates, for example, XYZ or CIELAB values. The Macbeth ColourChecker DC (the DC chart), has been widely used in previous studies for color characterization tasks (Burns and Berns, 1996; Finlayson et al., 1997; MacDonald and Ji, 2002). It consists of 232 monochromatic and chromatic matte patches and 8 glossy patches. Figure 4 shows the color distributions of the DC chart (232 matte patches) in CIELAB color space. Hence, both the self-developed “Qianlong Palette” color chart and the DC chart (excluding the 8 glossy patches) were selected as training datasets for silk fabric color prediction. In order to evaluate the model prediction of the color of silk fabrics, both the DC chart itself and the “Qianlong Palette” color chart were used as the testing dataset; for the “Qianlong Palette” color chart, that chart was used as the testing dataset. In addition, three degrees (first, second, and third order) of polynomial regression (PR) were used as the mapping methods of the camera model. The 1st PR has four terms in the model: R, G, B, 1; the 2nd PR has ten terms in the model: R, G, B, R^2^, G^2^, B^2^, RG, RB, GB,1; and the 3*^rd^* PR model includes twenty terms: R, G, B, R^2^, G^2^, B^2^, RG, RB, GB, R^2^G, R^2^B, G^2^B, RG^2^, RB^2^, GB^2^, R^3^, G^3^, B^3^,1. The predictive accuracy of the color characterization for different models was quantified by the mean color difference between the instrument measurement color (JETI spectroradiometer) and the model predicted color (converted from the camera RGB values) for all the testing color samples. CIELAB is used to predict color difference in this study under standard D65 lighting. The different training datasets and mathematical modeling methods were compared in terms of their predictive accuracy of the silk fabrics’ color.

#### Display color characterization

An EIZO color professional display (EIZO CG246, 120 cd/m^2^, 6500K, sRGB color space, gamma 2.2) was used to present the color images of the silk fabrics. Before the radiometric measurements for characterization, the monitor was placed where it would be used and then turned on for 1 h to warm up. The spatial independence and the channel independence were tested for accurate characterization using the JETI spectroradiometer. The GOG (gain-offset-gamma) model was then employed to carry out the display color characterize (Berns, 1996; Day et al., 2004; Xiao et al., 2011). The pure red, green, blue patch and a serious of gray scale patches, seen against a neutral background, were used as the training dataset for building the GOG model. The forty-two color patches from the “Qianlong Palette” color chart, again seen against a neutral background, were used as the testing dataset. The mean color difference between the instrument measurement and the model prediction was calculated to evaluate the accuracy of the display model.

#### Interface development

**FIGURE 5:**
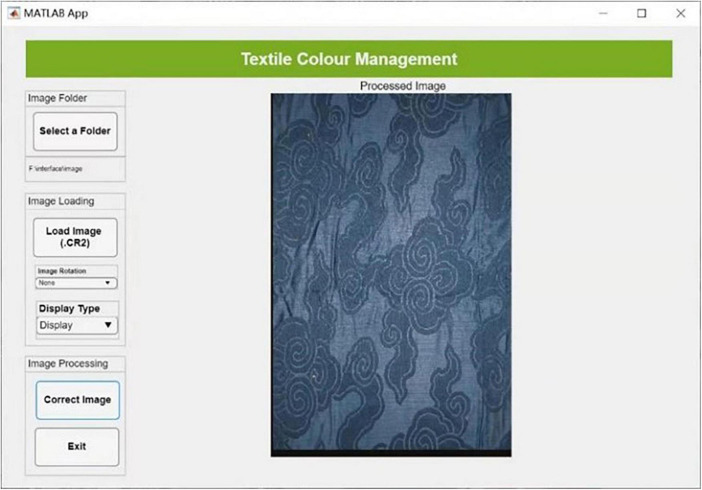
The self-programmed graphic user interface for textile color management.

After the color management was completed, a self-programmed graphic user interface was created, as shown in Figure 5 (also see Figure 2C). The interface was designed for automatically processing any new images of silk fabrics taken using the digital imaging system. The color characterization models of both the camera and the display with the highest precision of color reproduction were built into the interface. Therefore, any input of a RAW image file from the digital camera can be processed automatically and, after color correction, an output of the corresponding TIFF image file with the same resolution can be generated and saved. This is a high-quality digital copy of the cultural relics and is ready for use in different applications.

#### System validation

The performance of the digital archive system was tested and validated using six representative silk garments with different colors in the Ming and Qing Dynasties borrowed from the China National Silk Museum. The spectral reflectance of those silk garments was measured using a Konica Minolta CM700d spectrophotometer with a small aperture size (3 mm) and transformed to CIEXYZ tristimulus values. For each garment, a uniform color region was chosen, and three target measurement points were selected within the uniform color region. Color images of the silk fabrics were captured using the digital imaging system and then processed with the self-programmed graphic user interface to provide color correction. For the same three measurement points, the camera RGB in the fabric image was obtained and transformed to CIE XYZ tristimulus values using the proposed camera color characterization model. The mean CIELAB color difference between the instrument measurements and the image color predictions over three measurement points were calculated and referred as the objective measure of the system accuracy. Additionally, a subjective assessment was conducted to evaluate quality of color reproduction visually by two expects in textile cultural relics from the China National Silk Museum.

## Results

### Camera color characterization models

**FIGURE 6:**
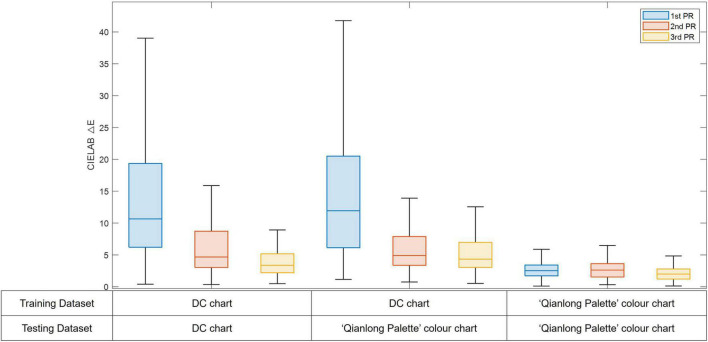
The distribution of CIELAB color difference across all testing samples in different camera color characterization models (The line inside of each box is the median ΔE*_ab_; the top and bottom edges of each box are the upper and lower quartiles, respectively; the whiskers are lines connect the maximum ΔE*_ab_ and the minimum ΔE*_ab_).

Nine camera color characterization models were evaluated including two training datasets (the DC chart and the “Qianlong Palette” color chart) and three mapping methods (1st PR, 2nd PR, 3rd PR). The results of all models were listed in Table 1 including the mean, minimum, and maximum CIELAB color difference across all testing color samples. The distribution of the color difference across all testing samples in different camera color characterization models shows in the box plots in Figure 6.

**TABLE 1:** The camera color characterization results.

Training dataset	Testing dataset	Mapping method	Mean ΔE*_ab_	Min. ΔE*_ab_	Max. ΔE*_ab_
DC chart	DC chart	1st PR	13.88	0.41	47.26
		2nd PR	6.06	0.36	33.22
		3rd PR	4.27	0.50	22.98
DC chart	“Qianlong Palette” color chart	1st PR	15.12	1.16	56.20
		2nd PR	5.86	0.75	15.93
		3rd PR	5.33	0.54	20.33
“Qianlong Palette” color chart	“Qianlong Palette” color chart	1st PR	2.86	0.11	13.17
		2nd PR	2.74	0.33	10.44
		3rd PR	2.13	0.12	6.68

The color reproduction accuracy for the silk fabrics has been significantly influenced by the training datasets and the mathematical mapping methods. Using both color charts, the 3rd polynomial regression shows the best predictive accuracy, followed by the 2nd and the 1st order polynomial regression. However, the influence of the training dataset was much larger than the influence of the mapping methods. Although the DC chart has been widely used in different applications, it fails when used to predict the silk fabric colors, with a best predictive accuracy of 5.33ΔE*_ab_. Use of the “Qianlong Palette” color chart leads to the best predictive accuracy with the 2.13ΔE*_ab_ when using the 3*^rd^* PR model and the worst predictive accuracy is 2.86ΔE*_ab_ when using the 1*^st^* PR. The accuracy of the camera color characterization can reach 2.13ΔE*_ab_ by selecting the third-order polynomial regression method and using the “Qianlong Palette” color chart to train the model.

### Display color characterization model

The spatial independence of the display was first assessed by measuring the CIELAB color difference between a white patch with a black surround, in the center of the display, and the same white patch with a white surround, and the color difference was found to be at 0.52ΔE*_ab_. The channel independence was assessed by the color difference between a full-field white and the prediction of the full-field white based on the sum of the tristimulus values of the full-field pure red, green, and blue, and the color difference was 0.75ΔE*_ab_. Both results indicated acceptable spatial and channel independence. The same forty-two colors from the “Qianlong Palette” color chart against a neutral background were used as the testing dataset to test the color performance of the display. The GOG model generated a mean color difference of 0.26 ΔE*_ab_ within the training dataset and 0.79ΔE*_ab_ for the testing “Qianlong Palette” colors. Because the GOG model gave a relatively good color reproduction performance, other models for display color characterization were not considered.

### System validation

The digital archive system was validated both objectively and subjectively using the real textile cultural relics. Six representative silk garments with different colors in the Ming and Qing Dynasties were borrowed from the China National Silk Museum. Table 2 shows the detailed information and corresponding images of all six silk garments. All the garments were digitally archived using the system described above and their high-resolution images were captured, processed, and finally displayed on the EIZO display. The CIELAB color difference between the instrument measurements and the system color prediction for each garment was calculated and listed in Table 2. The mean CIELAB color difference of the six garments was 2.53ΔE*_ab_ with a standard deviation of 0.33ΔE*_ab_, indicating an acceptable color accuracy. For subjective assessment, validation work was conducted in the China National Silk Museum by two experts in textile cultural relics. After the digital archive process, the images generated from the system were evaluated visually on the color professional display in a dark room. Both the processed images after color management and the raw images were assessed in terms of the color reproduction results comparing to the color of the real silk fabric garments. The two expects were both satisfied with the processed images and also acknowledged the value of the digital archive system with regard to the protection of the textile cultural relics.

**TABLE 2:** The results of color prediction accuracy of the six silk fabric garments.

Garment No.	Name	Images	Mean ΔE*_ab_
1	Stain with branch peonies pattern on camel ground in late Qing dynasty	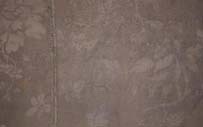	2.98
2	Floral roundel velvet on brown stain ground in late Qing dynasty	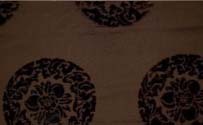	2.12
3	Colorful embroideries and flower patterns on azurite ground in Qing dynasty	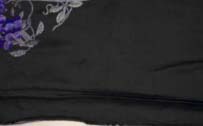	2.35
4	Clouds patterns on light blue ground in Qing dynasty	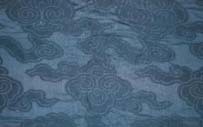	2.30
5	Colorful embroideries and flower patterns on red ground in Qing dynasty	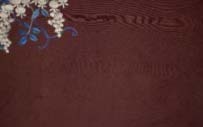	2.59
6	Gauze with ruyi(good luck)pattern in a group of four and clouds in Ming dynasty	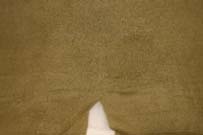	2.84

## Discussion

Chinese textile manufacture and dyeing techniques had a long history and reached a period of great prosperity during the Qing Dynasty. The court costume and accessories, especially the Imperial Chinese robes are representations of the finest level of textile production during that period. Research on those textile cultural relics is of great importance to the understanding and development of the textile history both in China and elsewhere in the world. Color was an extremely important element of the court costumes as the symbol of social hierarchy in that period, and the dress code was strictly governed, e.g., the bright yellow could only exist on the imperial lobes of the emperor and the empress, and various other colors were used on the robes of different members in the royal family (Han et al., 2018). Therefore, the color appearance of those silk fabrics is of great historical value and it’s crucial to show their real colors when they are viewed in both a physical museum and on an online museum.

The textiles are classified as the most light-sensitive museum artifacts, and they can only be exposed to a very low light level of less than 50 lux in the museum to avoid cumulative and irreversible damage that could be caused by light exposure (Illuminating Engineering Society [IES], 2017). Thus it is difficult to faithfully show the real color of the textile cultural relics under such a low light level and it is common practice to use a dim environment for textile exhibitions in the museum (Ballard et al., 2015). However, the digital museum and imaging technology provide a new opportunity to achieve accurate color reproduction of these cultural relics as part of a digital collection.

In this study, using the digital archive system, the textile samples were captured under a lighting system simulating standard illuminant D65 and, after color correction, the images reproduced the color under the standard lighting. In this way, not only would the audience have a better experience when viewing the colorful Imperial Chinese robes, but the real textile cultural relics could also be well protected without further light exposure.

In order to achieve accurate color reproduction, a “Qianlong Palette” color chart was specifically designed for color management of the Imperial Chinese robes and other silk fabrics in the Qing Dynasty. All the colors were selected based on the archeological documents and included different color series. The color ranges and distributions (Figures 1B,C) were the representations of the colors used for court costumes and accessories during the Qianlong period of the Qing Dynasty. In addition, a special silk material, plain crepe satin, and various natural dyestuffs, were used to simulate the ancient dyeing process and to mimic the appearance of silk fabrics in the Qing Dynasty as closely as possible. In the color characterization of any image processing system, a test chart is usually required. The Qianlong Palette’ color chart that was developed is a unique concept and its performance was tested and found to be superior over the more conventional DC chart. Considering the number of color patches are similar in the two charts, a possible reason for this finding is that the materials properties have influenced the predictive accuracy. The material properties, such as the gloss and the reflectance of the silk fabrics are very different to those of the color patches of the conventional color chart. Though the DC chart didn’t give very good results when predicting its own colors due to the large color differences produced by all the black patches on the DC chart, the results were slightly better than its prediction of the textile sample colors. The color characterization model based on using the “Qianlong Palette” color chart as the training set gave good prediction to the new silk fabric samples of the real textile garments. Thus, a color chart with a similar material is to be much preferred as the training target for color prediction of the silk fabrics. The Qianlong Palette’ color chart developed in this study is of great value in the accurate color reproduction of the silk fabrics in the Qing Dynasty and can be widely used in the future for the color correction of further textile cultural relics and other textile materials.

Previous research has shown that using higher order polynomial regression for camera characterization models generates better performance in terms of color prediction (Hong et al., 2018). Consistent with these previous findings, our results also indicate that a higher order polynomial regression model is preferred when used on images of silk fabric samples. However, for the color prediction of silk fabrics, the impact of the mapping method is weaker than the impact of the training dataset. Using the “Qianlong Palette” color chart as the training dataset, even the lower order polynomial regression model was able to generate acceptable accuracy for the color prediction of the silk fabrics. Generally, the results showed that both training dataset and mapping methods affect the predictive accuracy, and the “Qianlong Palette” color chart could largely improve the color prediction of the camera characterization models.

The digital archive system relies on digital imaging technology, produces high-quality images with accurate color reproduction, and has significance in both the protection of cultural relics and the development of online museums. The system also shows great potential in supporting digital collections or exhibitions, helping the spread of traditional Chinese crafts, and improving communication of textile cultural heritage worldwide.

## Conclusion

This study has developed a digital archive system for accurate color reproduction of imperial silk robes from the Qing Dynasty. The newly designed “Qianlong Palette” color chart exhibits superior performance over use of the conventional color chart in effectively color managing an imaging system to predict the color of the silk fabrics. The self-programmed graphical user interface for image color management can serve as a powerful tool to truly reproduce the color of various silk fabric relics in museums in the future, and digitally archive those valuable cultural relics for different uses.

## Data availability statement

The datasets presented in this article are not readily available because the original contributions presented in the study are included in the article/supplementary material, further inquiries can be directed to the corresponding author. Requests to access the datasets should be directed to YL, sdyl@leeds.ac.uk.

## Author contributions

All authors listed, have made substantial intellectual contribution to the work, and approved it for publication.
